# The S1 Subunit of the SARS-CoV-2 Spike Protein Activates Human Monocytes to Produce Cytokines Linked to COVID-19: Relevance to Galectin-3

**DOI:** 10.3389/fimmu.2022.831763

**Published:** 2022-03-22

**Authors:** John T. Schroeder, Anja P. Bieneman

**Affiliations:** The Department of Medicine, Division of Allergy and Clinical Immunology, Johns Hopkins Asthma and Allergy Center, Johns Hopkins University, Baltimore, MD, United States

**Keywords:** inflammation, lectin, cytokine, virus, innate immunity, dendritic cell, basophil

## Abstract

Coronavirus disease 2019 (COVID-19), caused by the severe acute respiratory syndrome coronavirus 2 (SARS-CoV-2), rapidly evolved into a pandemic –the likes of which has not been experienced in 100 years. While novel vaccines show great efficacy, and therapeutics continue to be developed, the persistence of disease, with the concomitant threat of emergent variants, continues to impose massive health and socioeconomic issues worldwide. Studies show that in susceptible individuals, SARS-CoV-2 infection can rapidly progress toward lung injury and acute respiratory distress syndrome (ARDS), with evidence for an underlying dysregulated innate immune response or cytokine release syndrome (CRS). The mechanisms responsible for this CRS remain poorly understood, yet hyper-inflammatory features were also evident with predecessor viruses within the β-coronaviridae family, namely SARS-CoV-1 and the Middle East Respiratory Syndrome (MERS)-CoV. It is further known that the spike protein (S) of SARS-CoV-2 (as first reported for other β-coronaviruses) possesses a so-called *galectin-fold* within the N-terminal domain of the S1 subunit (S1-NTD). This fold (or pocket) shows structural homology nearly identical to that of human galectin-3 (Gal-3). In this respect, we have recently shown that Gal-3, when associated with epithelial cells or anchored to a solid phase matrix, facilitates the activation of innate immune cells, including basophils, DC, and monocytes. A synthesis of these findings prompted us to test whether segments of the SARS-CoV-2 spike protein might also activate innate immune cells in a manner similar to that observed in our Gal-3 studies. Indeed, by immobilizing S components onto microtiter wells, we show that only the S1 subunit (with the NTD) activates human monocytes to produce a near identical pattern of cytokines as those reported in COVID-19-related CRS. In contrast, both the S1-CTD/RBD, which binds ACE2, and the S2 subunit (stalk), failed to mediate the same effect. Overall, these findings provide evidence that the SARS-CoV-2 spike protein can activate monocytes for cytokines central to COVID-19, thus providing insight into the innate immune mechanisms underlying the CRS and the potential for therapeutic interventions.

## Introduction

The Coronavirus disease 2019 (COVID-19) pandemic, caused by the severe acute respiratory syndrome coronavirus 2 (SARS-CoV-2), has caused devastation worldwide with massive health consequences that continue to spawn enormous socioeconomic and political issues. While vaccines have had clear beneficial impact, COVID-19 cases, and a proportional number of deaths, continue to swell. And, as variants of the virus concurrently arise, so likely the need for updated vaccines and/or other preventative measures.

As with its predecessors within the Coronaviridae family, including SARS-CoV and the Middle East Respiratory Syndrome (MERS)-CoV, infection with SARS-CoV-2 is commonly linked to the development of acute respiratory distressed syndrome (ARDS) (1, 2). ARDS is the life-threatening condition involving a leakage of fluid into the lung that is most often responsible for the mortality seen in severe COVID. In addition, studies continue to reveal evidence for a dysregulated hyper-inflammation, or cytokine release syndrome (CRS) that is thought to contribute to acute lung injury and development of ARDS (3, 4). Among the cytokines over-expressed in COVID-19 are those generally linked to innate immunity, including pro-inflammatory cytokines (e.g. IL-6, TNF-α, IL-1β), chemokines (e.g. CXCL10/IP-10, CCL2/MCP-1, CCL3/MIP-1α, CCL4/MIP-1β, and IL-8), immunoregulatory cytokines (e.g. IL-10, TGF-β), and growth factors (G-CSF) (5–11). Studies are also emerging with evidence that several of these cytokines associate with and/or are predictive of severe COVID, with IL-6, CXCL10/IP-10, and IL-10 most often cited (7, 9, 10, 12, 13). Likewise, several studies point to various innate immune cells – many of which are well known for producing these cytokines– to be hallmark in the lung inflammation associated with COVID, with monocytes and macrophages most often implicated in the underlying pathogenesis of the disease (14–17). Yet, despite the mounting reports, there remains a poor understanding of the exact mechanism(s) underlying the dysregulated innate immune response and CRS associated with COVID-19.

Like SARS-CoV-1, SARS-CoV-2 uses Angiotensin-converting enzyme 2 (ACE2) as its major receptor to infect host cells (namely epithelial cells), which is mediated *via* the virus’ envelope-anchored spike glycoprotein (S). The mature S glycoprotein is a heavily glycosylated trimer, with each protomer composed of 1260 amino acids (residues 14-1273). The S1 subunit is composed of 672 amino acids (residues 14-685) and organized into four domains: an N-terminal domain (NTD), a C-terminal domain (CTD), which is also known as the receptor-binding domain (RBD), and two subdomains (SD1 & SD2). A transmembrane S2 subunit forms the stalk and is composed of 588 amino acids (residues 686-1273) (18, 19).

Within the NTD of SARS-CoV-2 (and other β-coronaviruses) is a region often referred to as the “galectin-fold”, given its high degree of structural homology to that of human galectin-3 (Gal-3) (20, 21). Because of this remarkable similarity, it has been proposed that the S1-NTD of SARS-CoV-2 may very well act like Gal-3 and that this might explain, in part, the immunological sequelae observed in COVID-19 (22, 23). Indeed, intracellular Gal-3 has been linked to immune cell activation, namely that of monocytes/macrophages (24). We also recently reported evidence that epithelial cell-associated Gal-3 (EC-Gal-3) can activate a variety of innate immune cells to produce pro-inflammatory cytokines (25–27). In particular, we showed the activation of human dendritic cells (DC) and monocytes, demonstrating that these cells produced high levels of IL-6 and TNF-α –two hallmark cytokines in COVID-19-associated CRS (27).

A synthesis of the above observations prompted us to test whether portions of the SARS-CoV-2 spike protein might also activate innate immune cells in a manner similar to that observed in our Gal-3 studies. Indeed, by immobilizing subunit components onto microtiter wells, we show that the S1 subunit (and likely the NTD portion) activates human monocytes to produce a near identical pattern of cytokines to that observed in COVID-19-related CRS. Other regions of the spike protein, such as S1-CTD/RBD, which binds ACE2, or the S2 subunit (stalk), failed to activate monocytes. Overall, these findings provide novel evidence that the S1 subunit of the SARS-CoV-2 spike protein directly activates monocytes for cytokines central to COVID-19-related CRS, with mechanistic implications fundamental to the pathogenesis of the disease.

## Materials and Methods

### Special Reagents, Buffers, and Media

The following reagents were purchased: crystallized human serum albumin (Calbiochem-Behring Corp, La Jolla, CA); PIPES, FCS, and crystallized BSA (Sigma-Aldrich, Allentown, PA); gentamicin, IMDM, and nonessential amino acids (Life Technologies, Inc, Grand Island, NY); Percoll (Pharmacia Biotec, Inc., Piscataway, NJ); rhIL-3 and the following recombinant SARS-CoV-2 Spike protein subunits: 1) S1/S2 “active trimer” (cat. # 10549-CV) consisting of a.a. 16-1211 and made resistant to Furin cleavage, yet capable of binding ACE2; 2) S1-RBD (cat. # 10500-CV) consisting of a.a. 319-541 and capable of binding ACE2; S1 (cat. # 10569-CV) consisting of a.a. 16-681, and S2 (cat. # 10594-CV) consisting of a.a. 686-1211. All were c-terminal His-tagged, HEK cell-derived, and contained no detectable endotoxin (R&D Systems, Minneapolis, MN). Some experiments used another S1 subunit (cat. # REC31806) containing a.a. 1-674) –also HEK cell-derived and with no detectable endotoxin yet Fc-tagged (The NativeAntigen Co., Oxfordshire, UK). All PIPES-containing buffers used in this study (e.g. 1x PIPES, PIPES/albumin/glucose –PAG, and PAG-EDTA) were made from a 10x solution, as previously described (27, 28). C-IMDM consisted of IMDM supplemented with 5% FCS, non-essential amino acids, L-glutamine, 10 μg/ml gentamicin, pH 7.2-7.4.

### Coupling of Recombinant SARS-CoV-2 Spike Protein Components to Microtiter Plate Wells

Recombinant SARS-CoV-2 spike protein components were coupled to polystyrene microtiter plate wells (ThermoFisher, Grand Island, NY). In brief, wells immediately received 0.100 ml of a 5 μg/ml solution after preparing in carbonate buffer (ThermoFisher, Grand Island, NY). Plates were covered and placed at 4°C for overnight. Within ~30 min. of initiating cell culture, the contents of each well was aspirated, with wells then washed three times using 0.250 ml 1x PIPES per wash. After the final wash, each well immediately received 0.100 ml C-IMDM before adding cells and stimuli for cell culture, as described in detail below. In the experiments using galectin-3-binding protein (LGALS3BP) as a reagent to block S1-induced monocyte activation, washed wells first received 0.200 ml PAG buffer to which 0.050 ml of serially-diluted solutions of 5x LGALS3BP (also in PAG) were immediately added. These plates were then incubated at 37°C, 5% CO_2_ for 1 hr. before transferring to 4°C until used for cell culture (~3h total). At that time, each well was again washed 3x with 1x PIPES (0.250 ml per wash) before adding 0.100ml C-IMDM to initiate set-up for cell culture.

### Isolation of Basophils, Monocytes and DC Subtypes From Blood

Basophils, monocytes and DC subtypes were prepared from residual TRIMA cassettes from anonymous subjects undergoing platelet pheresis. In some instances, venipuncture was performed on consenting adults (age range, 21-65 years) using a protocol approved by the Johns Hopkins University Institutional Review Board. Subjects were selected regardless of allergic status. Buffy-coats from both specimen sources were subjected to double-Percoll density centrifugation, which produces both basophil-depleted cell (BDC) and basophil-enriched cell (BEC) suspensions, as described (28). Basophils were purified from BEC suspensions by negative selection using an antibody cocktail & microbeads (StemCell Technologies, Vancouver, Canada, cat# 14309-A01P), and collecting the flow-thru from magnetized LS columns (Miltenyi Biotec, Gaithersburg, MD), as described in detail (28). Basophil purities ranged between 98% and >99%, as assessed by Alcian blue staining. The BDC suspensions were washed 4x to remove platelets before preparing monocytes and DC subtypes. Monocytes were prepared using CD14^+^ selection by collecting those binding to magnetized LS columns (Miltenyi), Monocyte suspensions regularly exceed 95% purity when prepared in this manner, as assessed by flow cytometry. The monocyte-depleted flow-thru cells were then partitioned to separately isolate pDC and mDC using negative selection protocols (StemCell Technologies, Vancouver, Canada). The few numbers of DCs isolated did not always allow for flow cytometric analysis, but previous studies indicate purities in the range of 50-90%, based on CD123^+^BDCA2^+^ (pDC) and BDCA1^+^ (mDC) staining (27).

### Co-Culture Conditions

All cultures to induce cytokine production by basophils, monocytes and DC subtypes were done in a manner similar to that previously described (26, 27). In brief, cells were suspended in C-IMDM such that 2x10^4^ (DC and monocytes) and 1x10^5^ (basophils) were added in 0.050 ml volumes to flat-bottom wells (96-well plates) pre-coated with spike protein components, and with all wells containing 0.100 ml C-IMDM. Immediately after adding cells, 0.050 ml of 4x the final IL-3 concentration (or medium alone) was added and the cultures incubated as indicated at 37°C, 5% CO_2_. Supernatants were harvested after 20h unless otherwise indicated and tested for cytokine secretion.

### Cytokine Measurements

Supernatants were analyzed for cytokine content using Bio-Plex plates capable of simultaneously measuring 27 cytokines in a 0.050 ml volume using Luminex technology (Bio-Rad, Hercules, CA). Assays were performed according to the manufacturer’s specifications and included standard curves for each cytokine. Plates were analyzed using a Bio-Plex 200 instrument (Bio-Rad, Hercules, CA). Supernatants were additionally analyzed for IL-6 protein by ELISA (ThermoFisher. Grand Island, NY).

### Statistical Analysis

Statistical analyses were performed with Prism 7.0 software (GraphPad, Software, LaJolla, Calif.) Analyses were performed using multiple paired t-test analyses unless otherwise specified. Differences were considered statistically significant at a *P* value <0.05.

## Results

### S1 Subunit of SARS-CoV-2 Activates Human Blood Monocytes to Secrete Cytokines Linked to COVID-19

In testing whether recombinant components of the SARS-CoV-2 spike protein activate innate immune cells for cytokine production, we focused on the effects potentially seen with basophils, monocytes, and dendritic cell subtypes (pDC and mDC) –all freshly isolated from blood. These cell types were chosen because we have shown that all are activated by EC-Gal-3. And, since the S1-NTD of the spike protein expresses a “galectin-fold”, we hypothesized that each might likewise be stimulated. Two additional approaches were done for these experiments: 1) cultures were performed in microtiter plates pre-coated with spike protein components, since preliminary results indicated that proteins used in solution showed no to little capacity to stimulate cells (data not shown); and 2) we investigated the effects of co-stimulation with IL-3. Importantly, both *in vitro* culture strategies had proved instrumental is establishing the role of Gal-3 in activating these cells types (26, 27).

**Figure 1:**
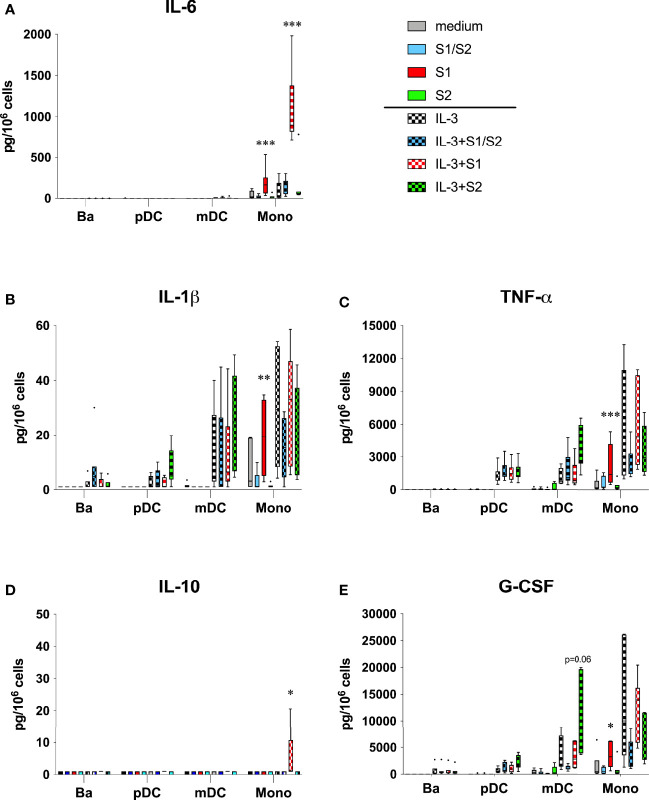
**(A-E)** Cytokines linked to COVID-19 are induced by the S1 subunit of the SARS-CoV-2 spike protein. Subunit components of the SARS-CoV-2 spike protein were passively absorbed onto polystyrene culture wells, as described in the Materials & Methods section. After overnight incubation at 4˚C followed with 3x washes, basophils (Ba), pDC, mDC, and monocytes (Mono) were then cultured as indicated in medium alone or with IL-3 added to 10 ng/ml. After 20h incubation, cell-free supernatants were harvested for analysis of the indicated cytokines using multiplex analysis. Box-Whisker plots (Tukey’s method) represent results from different donor cell preparations (n=7). Responses to spike protein components were tested for significance by comparing to medium/IL-3 controls. ***P<0.001, **P<0.01, *P<0.05.

We first investigated the effects on those pro-inflammatory cytokines that are hallmark in COVID-19. As shown in **Figure 1A**, effects were most evident with IL-6 production by monocytes. In particular, culture wells pre-coated with S1 induced 194 ± 64 pg/10^6^ monocytes vs. 41 ± 20 seen with medium alone. For comparison, monocytes averaged less IL-6 secretion in culture wells coated with either the S2 or the S1/S2 “active Trimer” components, with levels just 20 ± 8 and 21 ± 9 pg/10^6^, respectively. These amounts, however, were not significantly different from the IL-6 secreted in control cultures with medium alone. As predicted, the addition of IL-3 (10 ng/ml) augmented all responses and most significantly in culture wells coated with S1, where IL-6 levels averaged 12.5-fold more than those detected in the IL-3 controls (1104 ± 167 vs. 88 ± 48 pg/106, respectively). In contrast, IL-6 levels averaged just ~2-fold above the IL-3 controls for wells coated with S2 or the active Trimer (163 ± 104 and 148 ± 38 pg/10^6^, respectively), with neither significantly different. These IL-6 responses were not seen with any of the other cell types tested (basophils, pDC, or mDC), where levels mostly went undetected.

With results signifying that the S1 component of the spike protein activates monocytes for IL-6 secretion, additional analyses revealed a comparable pattern for other COVD-19 relevant cytokines produced in the same monocyte cultures. For example, IL-1β and TNF-α were both induced in culture wells coated with the S1 subunit, which were significantly higher than those measured in uncoated wells or wells containing either the S2 or S1/S2 components (**Figures 1B, C**). The addition of IL-3 did not augment these responses as it did for IL-6. Instead, IL-3 itself triggered monocytes to produce IL-1β and TNF-α. Whereas pDC and mDC also produced these cytokines, they primarily did so in response to IL-3 alone, with no evidence that any of the spike protein components directly acted on these DC subtypes. The S1 subunit also induced IL-10 in a couple of the monocyte cultures, although the levels were generally much lower and only evident when IL-3 was included. In contrast, none of the other spike protein components acted in a similar capacity to induce this cytokine (**Figure 1D**).

Several growth factors were among the panel of cytokines assayed by the multiplex analysis. As shown in **Figure 1E**, only the S1 unit mediated any significant affect by directly inducing G-CSF secretion by monocytes. There was a trend for increased production of G-CSF by mDC when cultured with S2 and in the presence of IL-3, yet this did not reach statistical significance. None of the spike protein components significantly impacted any other cell type for the production of the other growth factors investigated, which included FGF, PDGF, CM-CSF, or VEGF (**Figure S1**, online supplemental data).

As shown in **Figures S2**, **S3** of the online supplemental data, the spike protein components mediated little to no effect on most of the Th1 and Th2 interleukins analyzed, despite some predictable responses that lent validation to the multiplex analysis. For example, basophils cultured in IL-3 were clearly the predominant source of interleukin-13 among the four cell types investigated, as expected. However, these responses were not affected by any of the spike protein components analyzed (**Figure S3A**). Interestingly, the secretion of both IL-1ra and IL-15 was significantly affected, but not specifically by the S1 subunit. For example, IL-1ra was spontaneously secreted by monocytes in medium alone, yet this response was significantly reduced in culture wells coated with each of the three spike protein components (**Figure S2S**). Likewise, IL-15 was secreted by monocytes in response to IL-3, yet all three components significantly suppressed this response (**Figure S3E**).

### S1 Subunit of SARS-CoV-2 Activates Human Blood Monocytes to Secrete Chemokines Linked to COVID-19

**Figure 2:**
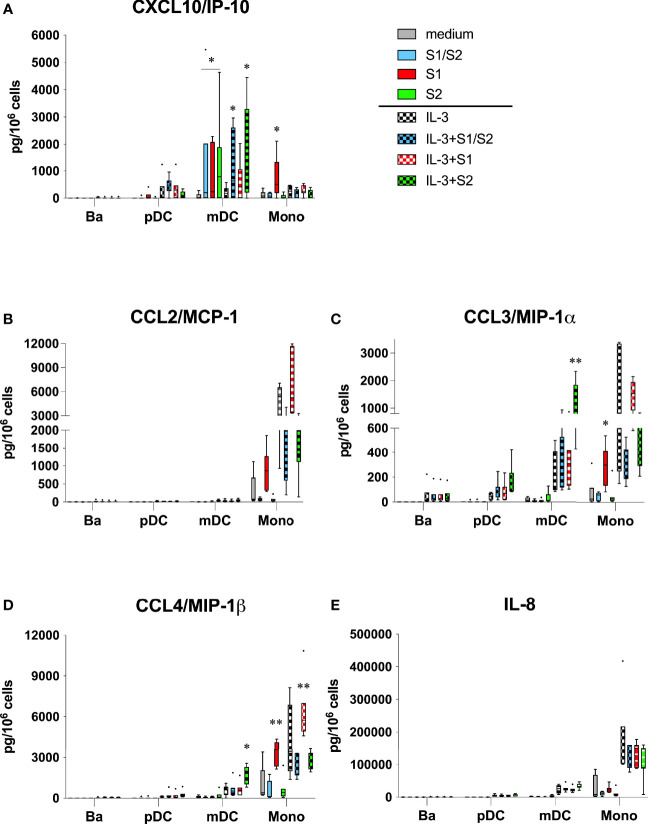
**(A-E)** Chemokines linked to COVID-19 are induced by the S1 subunit of the SARS-CoV-2 spike protein. The same culture supernatants described in **Figure 1** were also assayed for the indicated chemokines using multiplex analysis. Box-Whisker plots (Tukey’s method) represent results from different donor cell preparations (n=7). Responses to spike protein components were tested for significance by comparing to medium/IL-3 controls. **P<0.01, *P<0.05.

The S1 subunit also acted on monocytes to produce several chemokines that are prominent in severe COVID-19 (**Figures 2A–E**). In particular, CXCL10/IP-10, CCL3/MIP-1α, and CCL4/MIP-1β were all significantly induced in culture wells coated with S1, but not in culture wells containing S2 or the S1/S2 component. IL-3 augmented these responses for the latter two chemokines, although this was only significant for CCL4/MIP-1β. Oddly, both the S2 and S1/S2 components appeared to inhibit monocytes from producing these chemokines when compared to the controls, although levels were not significantly different. Likewise, a similar pattern was evident for CCL2/MCP-1, where S1 showed only a trend for inducing this chemokine vs. the medium control, yet significantly induced this this cytokine compared to the other spike protein components (**Figure 2B**). When used alone, the S1 subunit showed no capacity to induce any of these chemokines from the other cell types (basophils, pDC, or mDC). However, when combined with IL-3, the S2 subunit significantly induced both CCL3/MIP-1α and CCL/MIP-1β from mDC, but not from any other cell type. None of the spike protein components acted on the other chemokines measured in the multiplex analysis, including IL-8 (**Figure 2E**), CCL5/RANTES (**Figure S1E**), or CCL11/eotaxin (**Figure S3D**).

An overall summary of the monocyte cytokines significantly induced and/or affected by the S1 subunit is shown in **Table S1** of the online supplemental data. Included in these analyses are comparisons between values observed with S1 vs. those made in response to the S1/S2 and S2 components. In general, the latter two showed a trend to induce less cytokine, even when comparing to the medium and IL-3 controls.

### Activation of Monocytes by the S1 Subunit Does Not Track With the CTD/RBD Region Known to Bind ACE2

**Figure 3:**
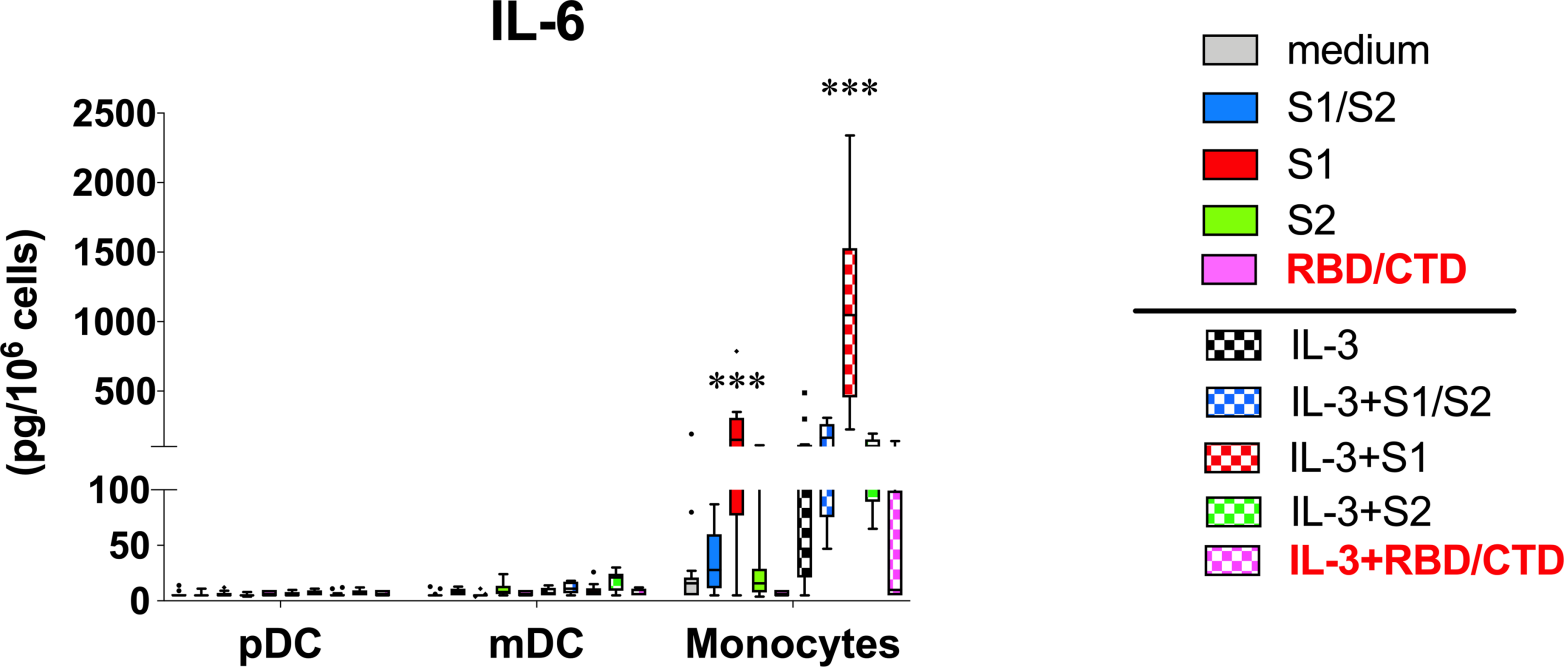
Capacity for S1 to activate monocytes for IL-6 secretion is lost using only the CTD/RBD region known to bind ACE2. Additional experiments (n = 5) were conducted like those described in **Figure 1** to test whether the S1-CTD/RBD region, which lacks the NTD, is capable of activating monocytes and DC. These experiments included using the full length S1 as a positive control. Since IL-6 measurements were made using ELISA, several supernatants from the multiplex analysis (**Figure 1**) were included for added validation. Box-Whisker plots (Tukey’s method) represent results from different donor cell preparations (n = 5-14). Responses to spike protein components were tested for significance by comparing to medium/IL-3 controls. ***P < 0.00001.

Structural analyses indicate that the so-called galectin-fold lies within the NTD of the S1 subunit (20). However, the S1 subunit used in the above cytokine experiments consisted of both the NTD and CTD/RBD (i.e. a.a. residues 1-681). Hence, it remained possible that the capacity of S1 to activate monocytes for cytokine secretion might still be attributed to the CTD/RBD region, and if confirmed, then a potential role for ACE2 despite this enzyme not typically found on immune cells. Therefore, additional experiments were conducted using another recombinant protein consisting of only the CTD/RBD region of S1 (a.a. residues 319-541). This component retains the capacity to bind ACE 2, as indicated by the data sheets provided by R&D Systems yet lacks the NTD region. For these experiments, we focused only on the capacity to induce IL-6, since this cytokine was readily secreted by the S1 subunit alone. However, cultures co-stimulated with IL-3 were also included with the goal of maximizing the IL-6 response. Measurements were performed using ELISA and included several of the previously analyzed specimens simply to lend validation of the IL-6 findings using the multiplex analysis. **Figure 3** shows that the same pattern of IL-6 was indeed detected as in the multiplex analysis and with comparable levels. However, the added experiments indicated little to no capacity for the CTD/RBD component to induce IL-6 from monocytes (used alone or with IL-3), despite robust responses from the same donor cells when using the full length S1 subunit that additionally contains the NTD.

### Galectin-3 Binding Protein Suppresses IL-6 Secretion by Monocytes Activated by the S1 Subunit

**Figure 4:**
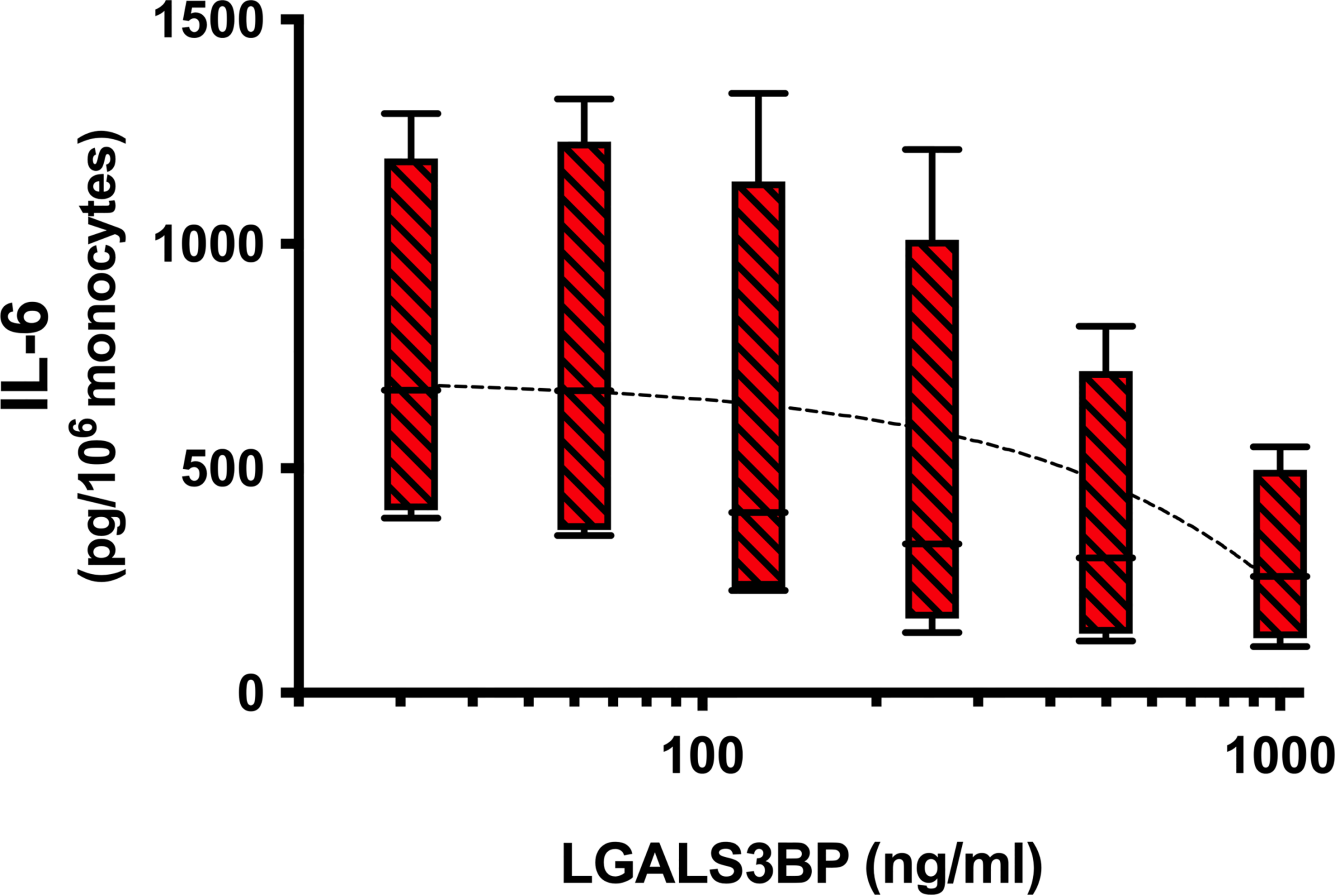
Galectin-3 binding protein (LGALS3BP) suppresses IL-6 secretion by monocytes activated by the S1 subunit. Increasing concentrations of LGALS3BP were added to wells pre-coated with the S1 subunit, as described in the *Materials & Methods*. After incubating, plate wells were washed 3x to remove excess LGALS3BP before adding monocytes and IL-3 for cell culture. Supernatants were harvested after 20h incubation and assayed for IL-6 by ELISA. Box-Whisker plots represent results from different cell preparations (n = 4), with a linear regression line shown (P = 0.012). IL-6 levels induced by S1 alone without LGALS3BP averaged 713 ± 258 pg/10^6^ monocytes (n = 4).

In a recent study conducted with the purpose of identifying novel serum proteins that bind/interact with the SARS-CoV-2 spike protein, the authors reported evidence that galectin-3 binding protein (LGALS3BP) was the top contender detected (29). Therefore, in a final set of experiments, we tested whether LGALS3BP might suppress the S1 subunit from activating monocytes for IL-6 secretion. To conduct these experiments, various concentrations of LGALS3BP were added to culture wells pre-coated with the S1 component. After incubating, the wells were then washed 3 times to remove any excess LGALS3BP. Again, IL-3 was added to maximize the S1-induced response. As shown in **Figure 4**, a consistent dose response suppression of the IL-6 produced by monocytes was observed with increasing amounts of LGALS3BP for an average inhibition of 59% (range 50-70%) observed at the 1μg/ml concentration (P=0.012).

## Discussion

The motivation for conducting this study evolved from two independent observations. The first originated from our work prior to the COVID-19 pandemic in which we showed evidence that epithelial cell-associated Gal-3 (EC-Gal-3) can effectively activate various innate immune cells for cytokine production (25–27). The second arose after the start of the pandemic upon learning that the SARS-CoV-2 virus contains a structurally relevant “galectin-fold” or pocket within the NTD of the S1 subunit of its spike protein –a structural feature first identified in the spike proteins of its predecessors (SARS-CoV-1 and MERS-Co) (20, 21). A synthesis of the two led us to hypothesize that the innate immune cytokine response (or CRS), which is most prominent in severe COVID-19, results, in part, from the S1-NTD of the spike protein mimicking the cytokine-inducing potential we had observed with EC-Gal-3. For further context, we have recently demonstrated that monocytes, and to a lesser extent DC subtypes, secrete IL-6 and TNF-α when co-cultured with A549 epithelial cells. However, these cytokine responses were eliminated upon knocking down Gal-3 expression in this adenocarcinoma cell line (27). We had also shown in earlier reports that IgE-expressing basophils produced IL-4 and IL-13 when co-cultured with EC-Gal-3 (26). Moreover, many of these EC-Gal-3-dependent cytokine responses were similarly replicated by culturing basophils, monocytes, and DC with microspheres coupled with rhGal-3 (MS-Gal-3). And, that IL-3 augmented Gal-3 dependent cytokine production by several of the innate immune cells, in particular basophils and pDC –those that bear the highest levels of IL-3R (CD123).

To address the belief that S1-NTD acts similarly to Gal-3 in promoting cytokine responses, we took the approach of using recombinant and endotoxin-free proteins that encompass various regions of the SARS-CoV-2 spike protein and that collectively span the entire ~1211 amino acid sequence. Importantly, with the exception of the S2 subunit (stalk), all of the recombinant proteins investigated possess the CTD/RBD, which, according to data sheets supplied by R&D Systems, enables functional binding to ACE2. Unfortunately, none of the proteins consisted only of the NTD. Nonetheless, of those investigated, only the S1 subunit, comprising the first ~615 amino acids and containing both the NTD and CTD, showed the capacity to induce cytokines from monocytes. In fact, this activity was observed using recombinant S1 proteins made by two different companies (see *Materials & Methods*). In contrast, both the CTD/RBD alone (a.a. 319-541) and the S2 subunit (a.a. 686-1211) failed to activate monocytes, thus implying the importance of the NTD –the region showing structural homology to Gal-3 (20, 21).

**Figure 5:**
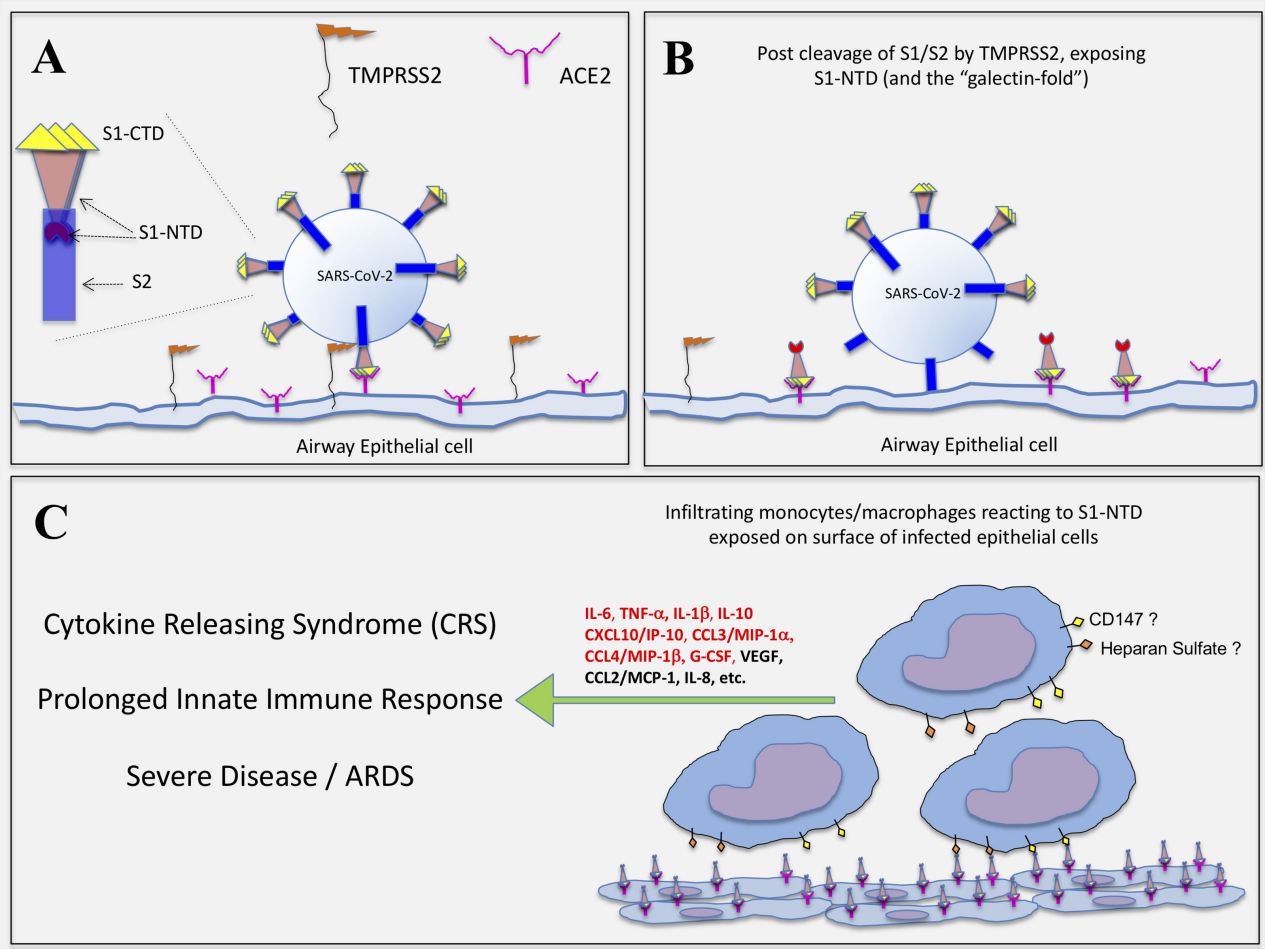
Hypothetical representation of how SARS-CoV-2 infection exposes the S1-NTD (and “galectin-fold”) on epithelial cells for potential activation of infiltrating monocytes. **(A)** SARS-CoV-2 infection of epithelial cells is initiated with the binding of S1-CTD/RBD to ACE2. The serine protease, TMPRSS2, expressed on host cells then cleaves the spike protein at the S1/S2 linkage. **(B)** The S2 subunit undergoes structural changes, serving first to anchor the virus and then facilitating its entry into the cell. It is proposed that the S1/S2 cleavage event simultaneously exposes the S1-NTD, which extends outward as the S1-CTD/RBD remains bound to ACE2. **(C)** Infiltrating monocytes/macrophages are then activated to produce COVID-related cytokines *via* cell surface glycoproteins (e.g. CD147) and/or polysaccharides (e.g. Heparan Sulfate) interacting with S1-NTD, which mimics EC-Gal-3. Those cytokines indicated in red type were significantly impacted by the S1 subunit in this study.

Most unexpectantly, the so-called S1/S2 “active trimer”, which encompasses nearly the entire spike protein sequence (a.a. 16-1211), failed to induce significant cytokine levels, despite including the S1-NTD. Exactly why this full-length spike protein lacked the capacity to activate monocytes remains unknown. However, we propose that in this form, the S1-NTD may not be properly exposed, perhaps even hidden, and thus unable to activate monocytes, as did the S1 subunit. In fact, R&D Systems specifies modifications to this protein making it resistant to furin (and perhaps other serine proteases), which means cleavage at the S1/S2 junction site is not possible. Accordingly, we further propose herein that a similar mechanism seems possible with the spike protein of live SARS-CoV-2 virus, at least during the course of infecting host cells. For instance, the virus attaches to ACE2 molecules expressed on host airway epithelial cells by utilizing the S1-CTD/RBD portion of its spike protein. However, it is known that viral entry is mediated by the S2 subunit, which is only primed and exposed after cleavage at the S1/S2 linkage site by the transmembrane serine protease 2 (TMPRSS2) and possibly by other host cell-associated proteases (30, 31). As a consequence, this cleavage event may serve to unmask the S1-NTD on the surfaces of infected cells, especially if the S1-CTD portion remains bound/anchored to ACE2, as conceptualized in **Figure 5**. If this mechanism is correct, it’s logical to hypothesize that the S1-NTD of the spike protein can then act in a manner similar to EC-Gal-3 and thus activate innate immune cells such as monocytes/macrophages that infiltrate virally infected lesion sites. Whether such a mechanism contributes to the CRS often seen in severe COVID-19 disease remains speculative at this time, yet the hypothesis is logical and thus requires further investigation.

Importantly, the mechanism proposed above (and in **Figure 5**) eliminates the prerequisite that immune cells express ACE2, which seems necessary if the virus was to promote cytokine production and dysregulation by directly infecting monocytes/macrophages *via* this receptor. However, there is currently little evidence in the literature to support ACE2 expression on human immune cells. Moreover, with the exception of the S2 protein, all the other spike protein components used in this study reportedly bind ACE 2. It therefore seems probable that all would have activated monocytes for cytokine production if binding to ACE 2 is critical for this response. In contrast, only the S1 subunit containing the NTD showed a capacity to activate monocytes. Naturally, our study did not use live virus, let alone test its capacity to bind ACE2, nor did we test whether it uses a variety of other receptors that have been proposed to facilitate its capacity to directly infect monocytes for induction of cytokines (14, 32–34). Therefore, we are not dismissing these possibilities yet only suggesting the above as another potential mechanism based on our findings using recombinant spike protein components and the resemblance of the S1-NTD region to that of human Gal-3.

Interestingly, of the innate immune cells co-cultured with plate bound S1 protein, only monocytes reacted by producing relevant cytokines. Basophils, pDC and mDC did not react to this spike protein subunit, as was predicted based on our prior work showing EC-Gal-3-dependent activation. While there is currently no precise explanation for these findings, one could argue that the S1-NTD, while closely resembling Gal-3, may not function to the full range of this lectin, especially given that we only tested recombinant protein. Moreover, Gal-3 is somewhat unique among the known mammalian galectins in that it can exist in many forms, ranging in structure from monomers to multivalent pentamers, each potentially having different binding affinities for many kinds of glycoproteins that contain the β-galactosides required for binding this lectin (35). For example, studies conducted ~30 years ago were among the first to show that Gal-3 binds IgE (hence, the early name of epsilon binding protein, ϵBP) (36). In fact, this partly explained its capacity to activate RBL cells (a mast cell line) for serotonin release (37), and more recently why basophils were required to express this immunoglublin when activated by EC-Gal-3 (26). However, Gal-3 has since been shown to bind many other non-IgE glycoproteins, including those for which the SARS-CoV-2 spike protein reportedly interact with, such as heparan sulfate (38) and CD147 (39). Although we did not explore the ligand(s) or receptor(s) on monocytes potentially binding the S1 subunit, it’s reasonable to hypothesize involvement of heparan sulfate and/or CD147 since both are reportedly expressed on monocytes (40, 41). Moreover, it seems possible that IL-3 may modulate expression of the putative receptor, since this factor generally augmented S1-induced cytokine secretion. Whether it acts *in vivo* to impact severe COVID-19 is presently unknown. Nonetheless, IL-3 is reported to promote the alternative activation of monocytes *in vitro*, thus it seemingly affects the plasticity of these cells (42). Future studies are required to determine the exact molecule/receptor on monocytes responsible for interacting with the S1 subunit to trigger cytokine secretion and whether IL-3 modulates its expression. Of particular importance, monocytes and macrophages are currently regarded to be the primary innate immune cells contributing to the CRS associated with COVID-19 (15–17). Therefore, the observation that monocytes were the only cells reactive to S1-NTD among those tested is consistent with this line of thought.

The pattern of monocyte-derived cytokines induced by the S1 subunit is among the more striking observations revealed in this study because the profile is remarkably similar to that implicated in the CRS associated with severe COVID-19. Again, IL-6 was the cytokine most consistently induced by the S1 subunit, which occurred regardless of whether IL-3 was added to augment the response. Likewise, IL-6 is perhaps the most consistently elevated cytokine associated with COVID-19 (7, 10, 13), which was the impetus for early trials testing whether blocking the activity of this cytokine (e.g. with anti-IL-6 receptor antibodies such as tocilizumab and sarilumab) might be useful in treating or preventing severe pneumonia in critically ill COVID-19 patients (43). Indeed, several studies have reported some efficacy in combating COVID-19 by blocking IL-6 activity (44, 45). To a lesser extent, TNF-α and IL-1β were other pro-inflammatory cytokines significantly induced by the S1 subunit and they too are cytokines that are generally increased in COVID-19. Interestingly, IL-10 has been linked to the CRS, but may play a pathological role by suppressing otherwise beneficial DC and T cell activity (46). While levels of this cytokine were generally low and detected in just a few of our experiments, its secretion was only evident when S1 was included in the culture (**Figure 1D**).

Several chemokines linked to COVID-19 were also significantly induced by the S1 subunit, including CCL3/MIP-1α, CCL4MIP-1β, and CXCL10/IP-10. All are implicated in playing a role in monocyte recruitment and are reportedly secreted by monocytes. Several studies, in fact, have reported CXCL10/IP-10 as a key marker of severe disease (9, 12). While our study also showed DC to be a source of this chemokine, only monocytes produced it in response to S1. In contrast, we observed no significant induction of CCL2/MCP-1 (only a trend) or IL-8 by S1, even though several studies report these chemokines increased in COVID-19 (47, 48). The same was true for VEGF (8). However, G-CSF was the only growth factor among those included on our multiplex panel that was significantly induced by S1 alone –it too is linked to COVID-19 (8).

As expected, no Th1/Th2 cytokines were induced by S1 or any of the spike protein components. Indeed, we know of no studies that consistently report increased level of any Th1/Th2 cytokines occurring with COVID-19. IL-2R levels are reported increased in the disease, but this cytokine was not among those investigated in our multiplex panel. Interestingly, our results showed that both IL-15 and IL-1ra, which were produced by monocytes when cultured in medium alone or with IL-3, were significantly decreased by all of the spike protein components tested. The bases for these latter findings remain unclear. Although, IL-1ra has been implicated in immune homeostasis, its suppression by the spike protein may thus help to promote dysregulation (1). Obviously, our *in vitro* cytokine findings do not definitively prove that the S1 subunit of the spike protein is responsible for inducing these in actual COVID-19 disease, but the comparison is quite striking and thus warrants further investigation.

To further support the notion that S1-NTD mimics Gal-3 in its capacity to activate immune cells, we demonstrated that Gal-3 binding protein, LGALS3BP, blocked (up to ~70%) the ability of the S1 subunit to activate monocytes. The rationale for conducting this set of experiments was two-fold. First, LGALS3BP is long known to interact with Gal-3, hence the name given to this 90kD protein. Therefore, we reasoned that it should also interact with S1-NTD, given the high degree of structural homology of this component with that of Gal-3. Second, a recent study reported evidence that recombinant SARS-CoV-2 spike protein, when added to serum/plasma specimens, specifically interacted with LGALS3BP, as determined by analysis using mass-spectrometry (29). Importantly, in conducting the experiments herein, we added increasing concentrations of LGALS3BP to wells pre-coated with the S1 subunit. After incubating, the wells were then washed extensively to remove any unbound LGALS3BP. Thus, not only was residual unbound protein removed prior to cell culture, which seemingly reduced any chances of LGALS3BP directly interacting with monocyte, its apparent interaction with plate bound S1 seemed stable enough to suppress monocyte activation. Overall, these results support the concept that the S1-NTD of the SARS-CoV-2 spike protein does, indeed, act like Gal-3.

In conclusion, the COVID-19 pandemic caused by the SARS-CoV-2 virus has to date claimed the lives of some 5.7 million worldwide, with over 900,000 of those occurring here in the United States alone (based on the JHU COVID-19 Dashboard). While vaccines continue to show remarkable efficacy in slowing these numbers and novel therapeutics continue to emerge, a better understanding of how this virus mediates immune dysfunction and the development of ARDS, remains poorly understood. Therefore, we propose that the findings presented herein provide insight into a potentially relevant mechanism –one in which the S1-NTD of the viruses’ spike protein (and likely that of other β-coronaviruses) mimics Gal-3 and the capacity of this lectin to modulate activation of innate immune cells, namely monocytes. Therefore, the development of therapeutics, such as Gal-3-like antagonists or neutralizing antibodies that target the S1-NTD of the spike protein, cannot be overstated in that they could prove efficacious in preventing prolonged innate immune dysfunction and onset of CRS leading to ARDS.

## Data Availability Statement

The raw data supporting the conclusions of this article will be made available by the authors, without undue reservation.

## Ethics Statement

The studies involving human participants were reviewed and approved by Johns Hopkins University IRB. Participants provided their written informed consent to participate in this study.

## Author Contributions

JS conceived the study, helped conduct experiments and wrote the manuscript. AB provided input regarding experimental design and conducted many of the experiments. All authors contributed to manuscript revision, read and approved the submitted version.

## Funding

Supported, in part, by Public Health Services Research Grants R01AI115703 and R01AI141486 to JS from the National Institute of Allergy and Infectious Diseases, National Institutes of Health (NIAID, NIH).

## Conflict of Interest

The authors declare that the research was conducted in the absence of any commercial or financial relationships that could be construed as a potential conflict of interest.

## Publisher’s Note

All claims expressed in this article are solely those of the authors and do not necessarily represent those of their affiliated organizations, or those of the publisher, the editors and the reviewers. Any product that may be evaluated in this article, or claim that may be made by its manufacturer, is not guaranteed or endorsed by the publisher.
