# Supplementary material for: The S1 Subunit of the SARS-CoV-2 Spike Protein Activates Human Monocytes to Produce Cytokines Linked to COVID-19: Relevance to Galectin-3
Source: Front Immunol. 2022 Mar 22;13:831763. doi: 10.3389/fimmu.2022.831763 (PMC8982143; doi:10.3389/fimmu.2022.831763)
Supplement: Supplementary file 1 [file Image_1.pdf]

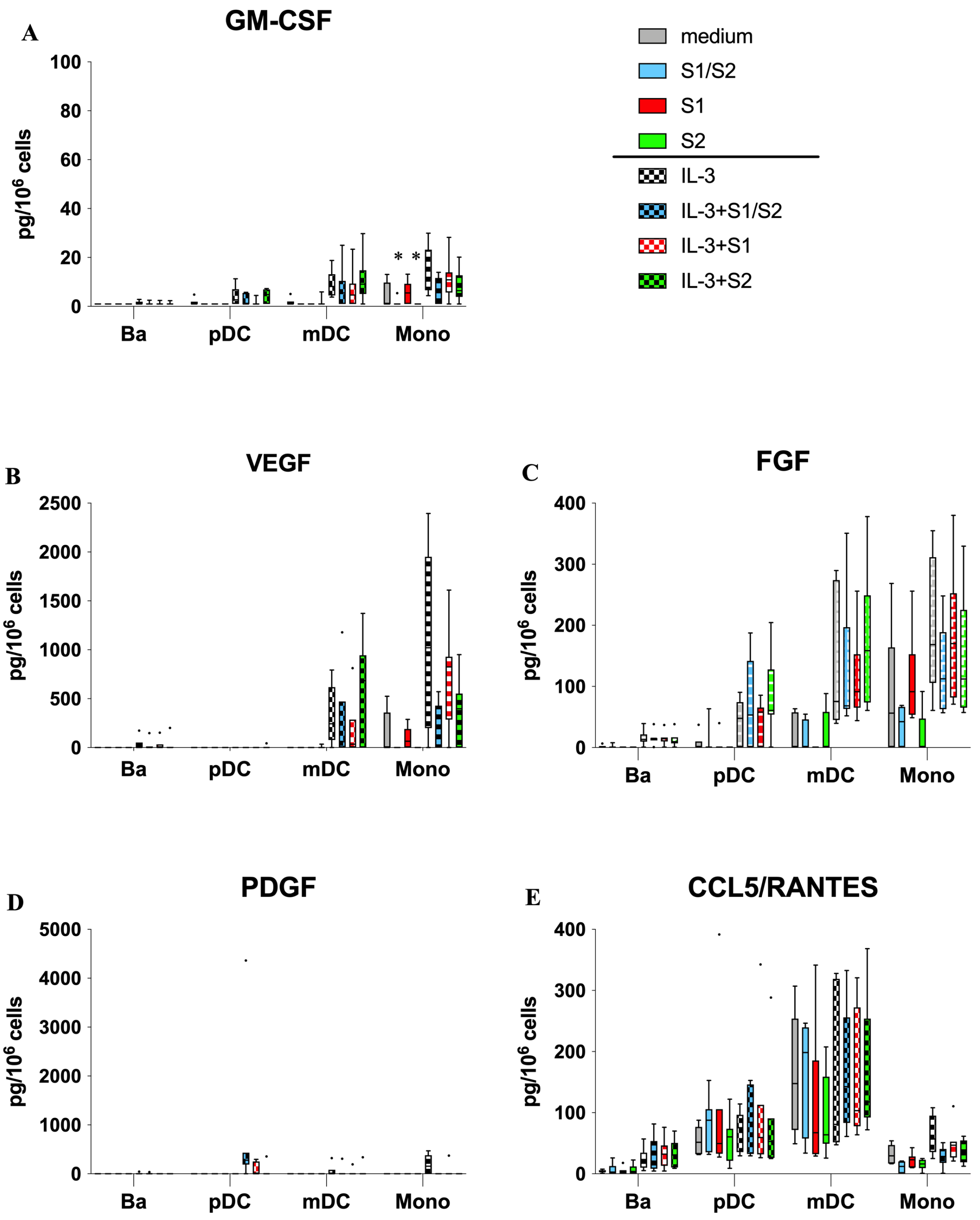


**Figure S1. Cytokine (growth factors & CCL5) responses to components of the SARS-CoV-2 spike protein.** Subunit components of the SARS-CoV-2 spike protein were passively absorbed onto polystyrene culture wells, as described in the Materials & Methods section. After overnight incubation at 4˚C followed with 3x washes, basophils (Ba), pDC, mDC, and monocytes (Mono) were then cultured as indicated in medium alone or with IL-3 added to 10 ng/ml. After 20h incubation, cell-free supernatants were harvested for analysis of the indicated cytokines using multiplex analysis. Box-Whisker plots (Tukey's method) represent results from different donor cell preparations (n=7). Responses to spike protein components were tested for significance by comparing to medium/IL-3 controls. *, $P<0.05$.
