# Supplementary material for: The S1 Subunit of the SARS-CoV-2 Spike Protein Activates Human Monocytes to Produce Cytokines Linked to COVID-19: Relevance to Galectin-3
Source: Front Immunol. 2022 Mar 22;13:831763. doi: 10.3389/fimmu.2022.831763 (PMC8982143; doi:10.3389/fimmu.2022.831763)
Supplement: Supplementary file 4 [file Table_1.pdf]

Supplemental Data

Table S1. Summary of P values for monocyte cytokines induced by S1 subunit

Cytokine	vs. medium		vs. S2		vs. S1/S2	
	-	(+ IL-3)	-	(+IL-3)	-	(+ IL-3)
IL-6	0.0005	<0.0001	0.0276	<0.0001	<0.0001	<0.0001
IL-1β	0.0066	NS	<0.0001	NS	<0.0001	NS
TNF-α	0.0005	NS	<0.0001	NS	0.0007	0.0035
CXCL10/IP-10	0.0415	NS	0.0208	NS	0.0284	0.0466*
CCL2/MCP-1	NS	NS	0.0039	0.0036	0.0041	0.0045
CCL3/MIP-1α	0.0171	NS	0.0047	0.0022	0.0018	0.0004
CCL4/MIP-1β	0.0042	NS	<0.0001	0.0015	<0.0001	0.0011
CCL5/RANTES	NS	NS	NS	NS	0.0416	NS
CCL11/EOTAXIN	NS	NS	NS	NS	NS	NS
IFN-γ	NS	NS	0.0313	0.0459	0.0330	0.0446
IL-1ra*	NS	NS	NS	NS	NS	NS
IL-2	NS	NS	NS	NS	NS	NS
IL-5	NS	NS	NS	NS	NS	NS
IL-8	NS	NS	NS	NS	NS	NS
IL-9	NS	NS	NS	NS	NS	NS
IL-10	NS	0.0112	NS	0.0060	NS	0.0060
IL-12	NS	NS	NS	NS	NS	NS
IL-13	NS	NS	NS	NS	NS	NS
IL-15*	NS	NS	NS	NS	NS	NS
IL-17	NS	NS	NS	NS	NS	NS
G-CSF	0.0110	NS	0.0218	NS	0.0069	0.0107
FGF	NS	NS	0.0128	NS	0.0329	NS
PDGF	NS	NS	NS	NS	NS	NS
GM-CSF*	NS	NS	NS	NS	NS	NS
VEGF	NS	NS	0.0369	NS	0.0369	0.0360

*****, cytokines that were significantly suppressed by spike protein components (see relevant panels in Figures S2 and S3). **NS**, not significant (multiple t-test).
